# Exercising is good for the brain but exercising outside is potentially better

**DOI:** 10.1038/s41598-022-26093-2

**Published:** 2023-01-20

**Authors:** Katherine Boere, Kelsey Lloyd, Gordon Binsted, Olave E. Krigolson

**Affiliations:** 1 Theoretical and Applied Neuroscience Laboratory, University of Victoria, STN CSC, PO Box 1700, Victoria, BC V8W 2Y2 Canada; 2 Faculty of Health, York University, 4700 Keele St, Toronto, ON M3J 1P3 Canada

**Keywords:** Neuroscience, Attention, Cognitive control, Human behaviour, Health policy

## Abstract

It is well known that exercise increases cognitive function. However, the environment in which the exercise is performed may be just as important as the exercise itself. Time spent in natural outdoor environments has been found to lead to increases in cognition similar to those resulting from acute exercise. Therefore, the benefits of both exercise and nature exposure suggest an additive impact on brain function when both factors are combined. This raises the question: what is the interaction between acute exercise and environment on cognition? We answered this question using electroencephalography to probe cognitive function using the oddball task before and after brief indoor and outdoor walks on 30 participants (average 21 years old, 95% CI [20, 22]). Our results demonstrate improved performance and an increase in the amplitude of the P300, an event-related neural response commonly associated with attention and working memory, following a 15-min walk outside; a result not seen following a 15-min walk inside. Importantly, this finding indicates that the environment may play a more substantial role in increasing cognitive function such as attention than exercise, at least in terms of acute exercise (i.e., a brief walk). With the world’s growing urbanization and the associated increase in sedentary time indoors, a deeper understanding of how these factors interact and influence cognition may be critical to combat adverse health effects.

## Introduction

It is well known that exercise generally enhances cognitive function^1–10^. However, the environment in which exercise is performed may be just as important as the exercise itself^11–13^. A growing body of research highlights nature's positive impacts on improving cognition and mental health^14–16^. Thus, with considerable evidence to support the benefits of exercise and being in nature, it is logical to expect that combining these two factors would lead to an ever-greater overall increase in cognition. Indeed, evidence supports this supposition: exercising outdoors in natural environments produces more benefits to the brain than exercising indoors^12,17–23^. Yet the findings are unclear for acute exercise durations under 20 min. This raises the question: How does the environment influence cognitive function for a brief exercise period? Gaining a deeper understanding of these underlying influences on the brain is important to combat the world’s growing urbanization and the associated increase in the amount of time spent indoors^24^.

In terms of cognition, acute outdoor exercise has primarily been found to enhance executive functions dependent on the prefrontal cortex, such as attention, working memory, and inhibitory control^1,3^. For instance, Bailey and colleagues^19^ found that participants who walked in an outdoor natural environment performed significantly better on a cognitive task—the Stroop task—than those who walked inside. These findings are further supported by Berman et al.^20^, who had participants complete a different cognitive task (the backwards digit span task) before and after a 35-min walk in a forest or an urban environment. In a key manipulation, the researchers induced cognitive fatigue in participants by having them complete a rigorous memory task before walking. Berman and colleagues’ results showed that on the cognitive task performed after the walk, participants who walked in the forest performed better than those who had walked in an urban environment suggesting that nature played an essential role in restoring the cognitive resources that were depleted by the memory task^20^. Together, these results provide evidence that outdoor exercise enhances executive function to a greater extent than indoor exercise.

Neuroimaging has provided key insight into the impact of acute exercise on the brain. Research with functional magnetic resonance imaging and functional near-infrared spectroscopy (fNIRS) has demonstrated that acute exercise drives improvements in cognition via increased cerebral blood flow (CBF) to the prefrontal cortex^25–32^. For example, Yanagisawa and colleagues used fNIRS to examine brain regions activated through acute exercise-induced enhancements in cognitive performance^27^. The researchers found that acute moderate exercise improved performance on the Stroop task and elicited increased activation in the dorsolateral prefrontal cortex- a region explicitly associated with executive function^27^. In addition, a range of neurotransmitters have been implicated in acute exercise's signalling pathways that induce positive cognitive and mood effects^1,3,28–32^. Specifically, several studies have found increases in dopamine, epinephrine, and norepinephrine in the prefrontal cortex post-exercise; all indicated to be involved in neuromodulating behaviours such as attention, reward, learning, and memory^29–33^. Collectively, these findings highlight the impact of acute exercise on the prefrontal cortex and indicate several mechanisms by which acute exercise influences the neurophysiology of this process.

Here we used neuroimaging to investigate the interaction of brief exercise and environment on cognition. Specifically, we utilized mobile electroencephalography (mEEG) to measure indices of cognitive performance prior to and after brief 15-min indoor and outdoor walks. Before and after each walk, participants completed a standard visual oddball task while mEEG data was recorded. Based on the abundance of literature indicating that exercise enhances cognitive performance^1–10^, we hypothesized that we would see an increase in the amplitude of the P300—a component of the human event-related brain potential (ERP) associated with working memory and attention—following exercise. Additionally, given the well-documented positive effects of nature on the brain^14–16^, we further hypothesized that the increase in the amplitude of the P300 would be greater following exercise outside than the following exercise inside.

## Results

To investigate the impact of walking location, we conducted a two (location: inside versus outside) by two (time: pre-test versus post-test) fully repeated measures analysis of variance on reaction time, accuracy, and P300 amplitude. See Table 1 for a summary of results.

**Table 1:** Summary statistical results.

	Inside		Outside	
	Pre	Post	Pre	Post
Mean reaction time (ms) [95% CI]	220.1 [210.2 230.1]	218.1 [207.0 229.3]	221.0 [210.0 229.3]	213.3 [203.4 223.7]
Mean errors made [95% CI]	0.8 [0.2 1.4]	1.2 [0.4 2.0]	1.4 [0.6 2.4]	0.7 [0.2 1.3]
Mean P300 amplitude (μV) [95% CI]	1.5 [0.8 2.2]	1.6 [0.8 2.4]	1.4 [0.5 2.4]	2.4 [1.6 3.1]

*95% CI* 95% confidence intervals.

### Reaction time

**Figure 1:**
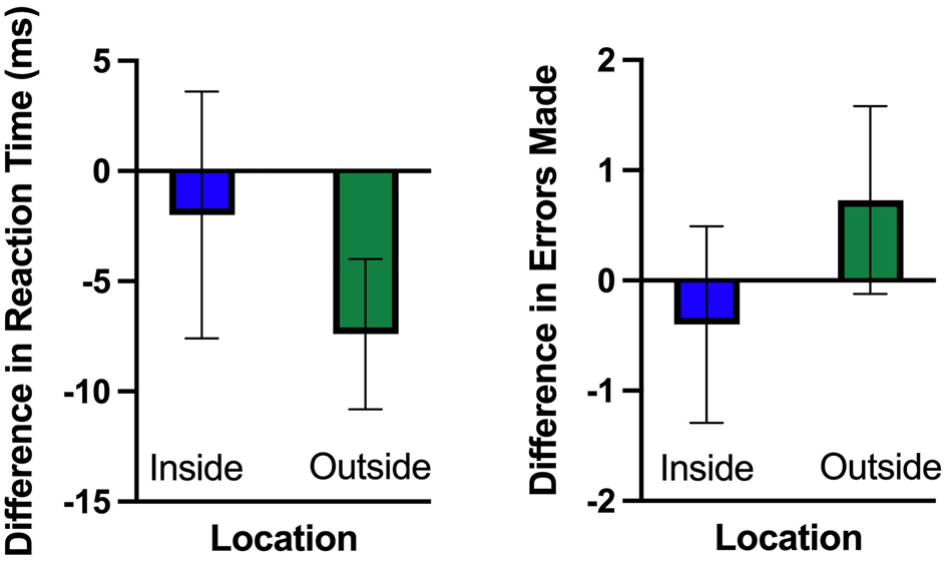
The difference in reaction time and accuracy on the oddball task between pre- and post- indoor and outdoor walk. *Value on plot* = *post walk score − pre-walk score*. Therefore, negative values indicate improved performance. Error bars reflect 95% within-subject confidence intervals.

An analysis of reaction time revealed a main effect for time (F(1,29) = 11.01, p = 0.002, η^2^ = 0.275) that showed that reaction decreased slightly following 15 min of walking (see Fig. 1). This was qualified by an interaction between walking location and time (F(1,29) = 3.95, p = 0.05, η^2^ = − 0.120). Decomposition of the interaction revealed that reaction time decreased after a 15 min outdoor walk (t(29) = 5.07, p = 0.000, d = 0.926) but not following a 15 min indoor walk (t(29) = 0.84, p = 0.409, d = 0.152). We did not find a main effect for walking location (p > 0.05).

### Accuracy

Our analysis of our accuracy data (number of errors made) revealed no main effects or interactions (F(1,29) = 3.59, p > 0.05, η^2^ = 0.10, see Fig. 1).

### P300 amplitude

**Figure 2:**
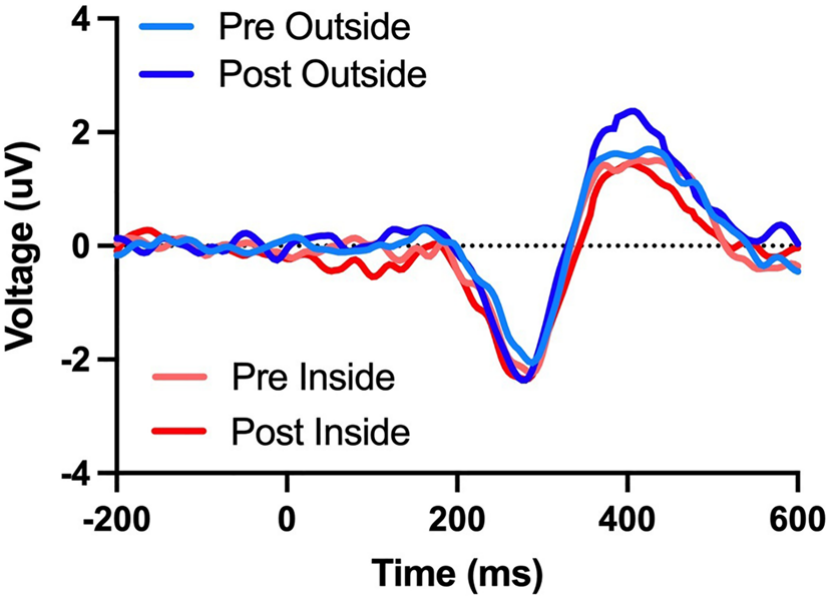
Grand average ERP difference waveforms for the visual oddball pre-test and post-test for indoor and outdoor walks. Note the P300 ERP component increased in the post-test following outdoor walks.

**Figure 3:**
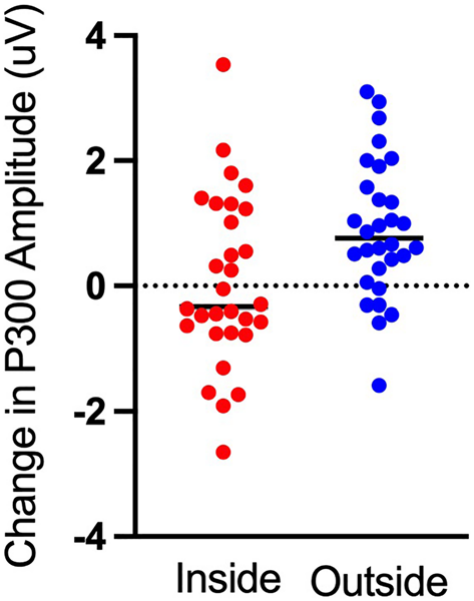
The difference in P300 peak amplitude between the post-test and pre-test for indoor and outdoor walks. Individual data are plotted for each participant.

Here, we found a main effect of time (F(1,29) = 12.40, p = 0.001, η^2^ = 0.299) that revealed an increase in P300 amplitude following 15 min of walking. This was qualified by an interaction between walking location and time (F(1,29) = 5.62, p = 0.025, η^2^ = 0.162). Decomposition of that interaction revealed no change in P300 amplitude following an indoor walk (t(29) = 0.23, p = 0.82, d = 0.032) but an increase in P300 amplitude following an outdoor walk (t(29) = 4.49, p < 0.001, d = 0.434, see Figs. 2 and 3). We did not find a main effect for walking location (p > 0.05).

## Discussion

In the present study, we examined how the walking environment—indoors or outdoors—interacted with acute exercise to impact cognitive function, specifically the oddball task and P300 amplitude. As predicted, exercise enhanced our measures of cognition, as evidenced by the rise in the amplitude of the P300 ERP component. However, this only occurred when the exercise took place outdoors. The main effect of exercise was extended to the behavioural results for reaction time, showing an overall decrease in reaction time post-walk. Yet again, the decrease in reaction times only occurred for outdoor walks. No difference was found in accuracy between groups, and we propose this is due to the ease of task difficulty. Our result is broadly consistent with a large body of research demonstrating that acute exercise enhances cognitive performance^1–3,5–7,9,10^. Further, previous EEG studies have shown that acute exercise increases P300 amplitude during task performance—a result associated with enhanced attentional processes and working memory in the pre-frontal cortex^34–38^. Our results align with notable findings that acute exercise improves brain function and is further supported by the positive effects found on task performance.

More importantly, we also found that outdoor exercise had an additional impact on cognitive attentional scores. Specifically, we found that the amplitude of the P300 ERP component was greater following a walk outside relative to a walk inside. In addition, reaction times were lower for outdoor walks than for indoor ones. This result is consistent with both attention restoration theory^13,39–41^ and previous findings supporting the idea that natural environments facilitate attention restoration during acute exercise^14–16,42^. The attentional restoration theory proposed by Kaplan^39^ posits that natural environments provide a sense of "being away" from routines and inducing "soft fascination." Rather than dominating one's attention, nature restores mental capacities and enhances cognitive performance^41,42^. Further, Kaplan and Berman^20^ proposed that natural environments restore directed attention, a shared resource that supports executive function in the prefrontal cortex.

Neurophysiologically, attention can be described as increased activity in a particular brain area involved in processing stimuli. As per the hemodynamic response, we assume increased activation signifies increased CBF in that specific brain region. For reference, the hemodynamic response is a homeostatic mechanism that replenishes the nutrients used by biological tissues by adjusting blood flow to areas of activity. Keeping in mind that this fresh, nutrient-rich blood is a limited resource—the attentional restoration theory implies that exposure to natural environments restores this mechanism by reducing unrequired increases in CBF. Regarding exercise’s effect on CBF, the reported results are mixed. We believe this is due to the amount of variability within exercise-based studies on the brain—as intensity, duration, and fitness are all significant factors that could influence the rate of CBF. However, when examining the effect of *acute low-moderate intensity exercise*, we identified abundant literature supporting that exercise increases CBF^1,2,5,7,25–28^. Indeed, with the lungs working harder during exercise, this oxygen surplus must be transported through the circulatory system. To facilitate this process, the body responds with a rise in heart rate and a widening of the arterial walls, thereby increasing blood flow globally, including to the brain. Taken together, we suggest that exercise increases CBF while the natural outdoor environments reduce and restore CBF mechanisms. Therefore, post-exercise and nature exposure, the effect of reduced blood flow to brain areas focused on irrelevant stimuli (brain areas not currently required) concurrently allows for increased blood flow and activation in areas pertinent to important stimuli (i.e., the task at hand).

An intriguing finding in our study is that we did not see a specific increase in the measured index of cognitive function following a brief walk indoors (i.e., we did not see an increase in P300 amplitude), nor did we find an increase in cognitive performance. This result conflicts with a significant meta-analysis by Chang and colleagues^3^ and fails to support the previously stated inference that brief exercise (less than 20 min) could promote cognitive function. The results imply that environmental location may facilitate attention restoration and improve indices of cognition without exercise. Such impacts of both nature and acute exercise could be synergistic; however, this begs the question, which plays a more prominent role in improving cognition? Although we cannot conclude this without separating exercise and environment, our results point to a more substantial role of environment in increasing cognitive function. Our findings suggest that if one has only 15 min to exercise^24^, performing it outdoors appears to have a greater effect on ERP indices of attention and working memory than indoors.

Notably, there are limitations to the conclusions we can draw from our findings. First, there is a lack of objective exercise intensity measurement. Although the participants were clearly explained, demonstrated, and paced with the desired walking intensity, no heart rate monitor was used. We recommend that all future studies introduce heart rate monitoring and rate of perceived exertion to further control variability and increase the validity of results. Second, previous research suggests that exercise duration must be over 20 min to affect cognitive performance positively^1,3,6^. Here, the duration of the walking protocol was decided from preliminary pilot testing, which recorded the time it would take a student to walk at a low/moderate intensity around a set track of 2 km. This resulted in a walking protocol of roughly 15 min. In addition, we specifically sought to investigate if the effects of this exercise differ when undertaken outside. Indeed, this time constraint may be why we did not find significant exercise-induced cognitive improvements. However, from this perspective, if the exercise duration is not long enough to influence cognitive performance, it is reasonable to deduce that the remaining variable—the environment—may be the driving force in increasing P300 amplitudes.

Considering the constraints of using mEEG over traditional large array systems is also essential. On mEEG, electrodes are placed in non-standard positions for observing “classic” ERP components such as the P300. Offline analyses of the oddball task typically would have focused on a central line electrode Pz as opposed to TP9 and TP10. Due to the MUSE device not having an electrode at Pz, we were forced to make this adaption. However, this is not a significant concern as previous work in our laboratory has demonstrated that although this produces a slightly different waveform, it still reveals clearly observable and quantifiable ERP components^47,48^. Second, due to time lags inherent with Bluetooth data connection and the inability to insert experimental event markers, we know there is a notable “jitter” in the EEG data. Yet, our previous work demonstrates that this does not prevent the recording or quantification of ERP components—it simply adds a delay which is accounted for in our analysis^47,48^.

In conclusion, we demonstrate that a brief walk outside results in a greater increase in cognitive function than a short walk inside. Given the continued growth in urbanization and a move to an indoor lifestyle, our results highlight the importance of spending time in nature, especially when exercising. Indeed, in a world where many people “hit the gym” before or after work or on their lunch break, our results suggest that these people would be better served by simply “getting outside”.

## Methods

### Study approval

All participants gave their informed written consent, approved by the Human Research Ethics Board at the University of Victoria (HREB: BC17-456). Human Research Ethics Board approved all experimental protocols at the University of Victoria (HREB: BC17-456). The experiment conformed to the ethical standards prescribed by the 1964 Declaration of Helsinki and subsequent revisions. All participants were given a comprehensive set of instructions regarding the procedure and tests, agreed both verbally and in the consent form to the testing procedures, and were given course credit in exchange for their participation.

### Participants

Participants in the present study were undergraduate, and graduate students at the University of Victoria, British Columbia, Canada (n = 32). Previous work in our laboratory^43^ conducted an ERP experiment with a sample size of 500 and found that detecting an ERP elicits a large effect size of 0.8, following recommendations from Cohen^44^. We conducted a power analysis for a repeated measures *t* test using this standardized effect size, an alpha of 0.05, and the desired power of 0.95, which yielded a prospective sample size of 25 participants. Moreover, our laboratory follows a protocol wherein ERP studies include a minimum of 30 participants, corresponding to a power of 0.99. It is important to note that participants with more than 50% of their data discarded due to excessive EEG artifacts were not included in our analysis. To avoid conducting underpowered research^45^, we kept testing participants until we had achieved our a priori set size of 30. Thus, the data presented here reflect the first 30 participants who met our EEG artifact criterion and resulted in the removal of two participants (n = 30; 21 females, average age: 21, 95% CI [20, 22]) Participants were asked to refrain from exercising and consuming alcohol for 24 h before testing. Further, each participant was instructed to abstain from eating or consuming caffeine two hours before testing. All participants had normal or corrected-to-normal vision and no known neurological impairments.

### Apparatus and procedure

Participants completed a standard visual oddball task on an Apple iPad (Apple Inc., Cupertino, CA, USA). At the same time, EEG data were recorded from a 2016 Muse EEG system (Interaxon Inc., Toronto, ON, Canada). The task and recordings were collected prior to and after 15-min indoor and outdoor walks. Walks were conducted on two separate days, on average 2 days apart and no more than 1 week apart (range = 1–7 days). The first participant walked inside on day one and outside on day 2. Subsequent participants alternated the walk locations on a participant-to-participant basis. The study was held at one of three times: 10:15 am, 12 pm or 1:45 pm—in an effort to limit the effects of the daily circadian rhythm.

Here, we choose the oddball task, rather than the previously used Stroop task^19,20^, to expand the current scientific literature on this topic. Both tasks are highly well-known in cognitive neuroscience, and both have been previously used to measure selective attention capacity and working memory^1–4,19,36,38^. Therefore finding the same results with a different yet equally valid task would only provide additional power to the result. During performance of the oddball task, participants saw a series of blue (RGB value = [0, 0, 255]) and green (RGB value = [0, 255, 0]) colored circles that appeared for 800 ms in the center of a dark gray background (RGB value = [108, 108, 108]) on the iPad screen. Before the onset of the first circle and between the presentation of subsequent circles, a black (RGB value = [0, 0, 0]) fixation cross was presented for 200 to 500 ms. Participants were not told the frequency of the blue and green circles: blue appeared less frequently (oddball: 30%) than green circles (control: 70%). Stimulus order was randomized, with the constraint that the stimulus presentation software ensured that no more than two infrequent (oddball) circles appeared consecutively. Participants were instructed to quickly press the bottom left or right of the iPad screen when they saw one of the blue circles (oddball) and not respond when they saw the green circles (control). The circles were presented for 800–1200 ms, and the trial ended automatically on oddball trials in which the participants did not respond. Each trial began with a fixation cross as soon as the previous circle disappeared. Participants completed four blocks of 100 trials.

After completing the pre-walk oddball task, participants completed a brief walk inside the Engineering Lab Building or outside the Alumni Chip Trail at the University of Victoria. This trail was a green and lush forested path around the campus. For both locations, the distance of the walk was 2 km. Participants were asked to walk at their normal pace—above leisurely but not hard breathing. For this reason, participants’ walk times varied between 14 and 17 min, with an average of 15 min. In addition, participants were asked not to speak to anyone, use their cell phones or listen to music for the entirety of the walk. A research assistant timed each walk and walked approximately 10 m behind the participant, pacing them to ensure they maintained the same walking intensity. Aside from the experimenter's directional instructions to ensure the participant kept a set pace, there was no conversation between the experimenter and the participant. After completing the walk, participants completed the oddball task again. All EEG recording was done in a quiet room within the Engineering Lab Wing building.

### Data acquisition

**Figure 4:**
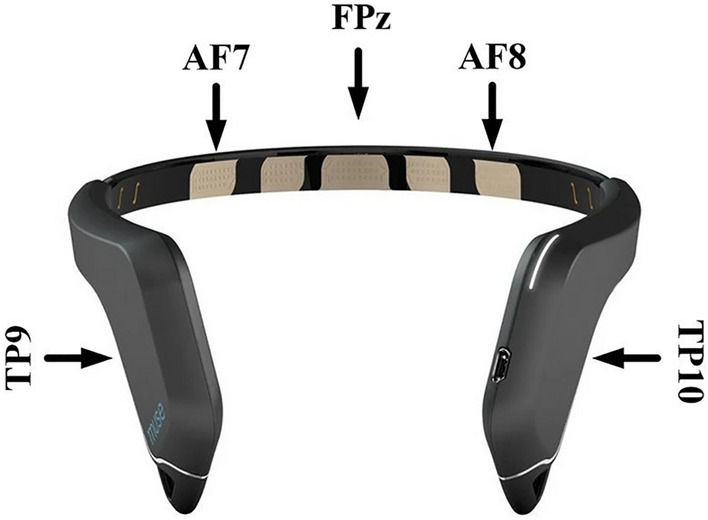
The 2016 Muse EEG system was made by InterAxon Inc with electrodes labelled AF7, AF8, TP9, and TP10. Reference electrode is labelled at FPz.

EEG data were recorded from a Muse EEG headband (Muse Version: 2016) sampling at 256 Hz (see http://www.choosemuse.com for full technical specifications, see Fig. 4). The Muse EEG system has electrodes located analogously to FPz, AF7, AF8, TP9, and TP10, with FPz utilized as the reference electrode during recording. EEG data were recorded, and the visual oddball task was performed with the PEER iOS application (https://brainwavesoftware.ca/). Note that the PEER application does not send event markers to the EEG headset as per a traditional ERP study^46^ but instead reads the EEG data with known Bluetooth lag and jitter. Specifically, we have written MATLAB code that allows two-way communication via the OSC protocol to send markers to mark a continuous EEG recording. As such, we “marked” the EEG data at the exact onset time when the circles were drawn. Due to the Bluetooth lag, the EEG samples corresponding to this point in time did not arrive for 18–20 ms on average with a jitter of approximately 5 ms^47,48^. It is important to note that this jitter only impacted the initial signal locking between the MUSE system and our software and did not vary over time. In addition, the random delays in the temporal onset of our data collection would have a Gaussian distribution, and thus the lags would average out.

Further, while the timing onset via Bluetooth is not “guaranteed,”—the order of data packets is (https://www.bluetooth.com/). Simply put, this means that all data points are guaranteed to be in the same order they were continuously collected in. As such, the averaging of temporal onset did not impact the present data as much as one might assume. Thus, the signal did not differ from trial to trial but from participant to participant. Signal quality was then inferred by examining the variance per second on each EEG channel, and data collection began when all channels had a variance per second less than 200. Finally, we have previously shown that this method still results in a reliable albeit diminished ERP response^47,48^ (see these papers for full details on Bluetooth timing issues and delay/jitter data).

### Data processing and analysis

Data were processed offline in MATLAB using EEGLAB^49^ and custom code. We did not re-reference the continuous EEG data offline as our ERP analysis focused on the two posterior Muse electrodes (TP9 and TP10) referenced when recording electrode FPz. Continuous EEG data were filtered with a dual-pass Butterworth filter with a 0.1–30 Hz passband and a 60 Hz notch filter. A preliminary analysis of the data revealed no lateralized effects; further, we wanted to improve the signal-to-noise ratio of the ERP measures^50^, so we created a pooled frontal and a pooled posterior virtual electrode by averaging across the frontal (AF7 and AF8) and the rear (TP9 and TP10) electrodes, respectively. Our ERP analysis only focused on the new average posterior virtual electrode based on our previous work^47,48^. We also chose not to analyze the ERP effects for the frontal ERP channels as re-referencing the EEG data mirrors the components given the high correlation between the Muse EEG channels—see https://www.krigolsonlab.com/muse-research.html for exploratory analyses examining this issue that provided the rationale for the choices we made here.

After filtering, epochs of data from 200 ms before to 600 ms after stimulus onset (oddball, control) were extracted from the continuous EEG data. Segments were then baseline corrected using the 200 ms preceding stimulus onset. An artifact rejection algorithm was then implemented to remove noise and blink artifacts; this procedure discarded segments with an absolute difference of more than 60 μV (on average: 30% [28.5, 31.5]). Segments were then averaged for each participant's oddball and control trials, and a difference waveform was constructed by subtracting the average control from the average oddball ERP waveform. Grand average ERPs were generated by averaging all conditional (oddball, control) and difference waveforms for each participant and the peak component latencies were identified (N200: ~ 200 ms; P300: ~ 400 ms). N200 and P300 ERP component amplitudes and latencies were quantified at the participant level by finding the local minimal (N200: 150–250 ms) and local maximal (P300: 400–600 ms) voltage amplitudes within the windows mentioned above around the grand average component peaks. ERP peak amplitude data were statistically analyzed using a two (walk location: indoor, outdoor) by two (time: pre-test, post-test) fully repeated measures analysis of variance. Post-hoc decomposition of the interaction was done via dependent samples *t* tests. Reaction time was calculated as the time it took participants to press the screen after the stimulus circle was presented. Accuracy was defined by the number of errors made during the task (responses to control stimulus or no response to oddball stimulus). Finally, mean data for ERP and behavioural data are presented with 95% confidence intervals.

## Data Availability

All processing scripts can be found at https://github.com/Neuro-Tools. The data that supports the findings of this study are available from https://osf.io/5m24j/.
